# Imagery retrieval may explain why recall of negative scenes contains more accurate detail

**DOI:** 10.3758/s13421-018-0876-7

**Published:** 2018-10-31

**Authors:** Chris R. Brewin, Kirsty M. R. Langley

**Affiliations:** University College London, London, UK

**Keywords:** Memory, Imagery, Valence, Recall, Pictures

## Abstract

We investigated whether the presence of imagery at retrieval was associated with the finding that negative pictures and scenes are recalled with greater perceptual detail. Participants were presented with 30 scenes taken from the International Affective Picture System that were rated either high or low on valence, but similarly on arousal. Recall was prompted with matched visual or verbal cues. During recall, participants reported any images that came to mind and rated them for vividness, whereas accuracy was rated independently. Imagery was described at test in response to over 60% of the stimuli. Whereas vividness was predicted by negative valence, images occurred more often in response to visual cues. The association of negative valence and visual cueing with better recall was observed only in the presence of reported imagery. These findings have important implications for models and experiments focusing on the recall and recognition of visual stimuli.

**Electronic supplementary material:**

The online version of this article (10.3758/s13421-018-0876-7) contains supplementary material, which is available to authorized users.

A finding with important practical and theoretical implications is that negatively, as compared to positively, valenced pictures and scenes are associated with superior recognition and sense of recollection (Ochsner, 2000), particularly with respect to their central visual details (Christianson & Loftus, 1991; Kensinger, 2009; Kensinger, Garoff-Eaton, & Schacter, 2006). Some reasons for this may be that negatively valenced scenes recruit more sensory processing areas at encoding (Kensinger & Schacter, 2008; Mickley & Kensinger, 2008) and that there is a greater recapitulation of this processing at retrieval (Bowen & Kensinger, 2017). Much less is known about the phenomenological processes operating at retrieval that correspond to or could mediate neural differences in the processing of positive and negative scenes. A number of lines of evidence pointing to the importance of imagery (Brewer & Pani, 1983; Brewin, 2014) suggest that the superior recall of visual detail accompanying negative stimuli could be a consequence of participants retrieving sensory images. In the present study, we report phenomenological data on imagery retrieval in the recall of visual scenes and investigate the extent to which such retrieval can explain the impacts of valence and different types of cue on the recall of positive and negative pictures.

Our capacity for long-term recall of visually detailed real-life scenes is very large (Brady, Konkle, Alvarez, & Oliva, 2008). The limit on the number of scenes greatly exceeds working memory constraints, although the quantity of information in each scene may be similar to that held in working memory (Brady, Konkle, Gill, Oliva, & Alvarez, 2013). More direct evidence that these representations play a functional role is provided by the observation that repeated recall of pictures produces hypermnesia, consistent with the presence of a persistent visual image that permits multiple opportunities for inspection and selection from the available information. Moreover, spontaneous visual memories of past autobiographical events are an everyday phenomenon (Brewin, Christodoulides, & Hutchinson, 1996) that can be produced in response to a wide variety of tasks, including generating word associations and passively viewing words and phrases (Berntsen, Staugaard, & Sørensen, 2013; Brewin & Soni, 2011; Mace, 2009; Schlagman & Kvavilashvili, 2008; Uzer, Lee, & Brown, 2012). Finally, individuals who report greater levels of visual imagery in general also show superior recall of pictures (Marks, 1973).

Less attention has been paid to how often images specific to the materials used are retrieved during the course of experimental tests of memory, but the fact that concrete words are recalled better than abstract words has strongly suggested a role for imagery in verbal learning (Paivio, 1969). The study of visual memory has established that the recall of previously encoded scenes is commonly accompanied by eye movements that are similar to those that occurred at encoding, and restricting these eye movements produces impairment in memory (Brandt & Stark, 1997; Johansson, Holsanova, Dewhurst, & Holmqvist, 2012). Diary studies have also shown that over several days, postexperiment memories of negative stimuli may return in the form of involuntary images, whether the stimuli are pictures (Bryant, McGrath, & Felmingham, 2013) or films (Brewin & Saunders, 2001; Ferree & Cahill, 2009; James et al., 2016). These studies are frequently used to test theories of posttraumatic stress disorder, a condition characterized by repetitive involuntary images of distressing scenes. The negative emotions associated with trauma are believed to be responsible for the persistence of these images (Brewin, 2014).

Although the participants in diary studies can often report the cues that triggered sudden recall of their autobiographical memories, there is controversy over which cues are most conducive to doing so (Mace, 2005). Where some studies (Berntsen & Hall, 2004) have shown that sensory cues are more likely to evoke them, others (Mace, 2004) have revealed that the majority of cues are language- or thought-based. In posttraumatic stress disorder, a condition characterized by the frequent retrieval of visual traumatic memories, it has been argued that sensory cues are likely to be more important than verbal cues (Brewin, Gregory, Lipton, & Burgess, 2010; Ehlers & Clark, 2000; Ehlers, Hackmann, & Michael, 2004). Sensory cues also appear to have an advantage because, in numerous types of experiments, greater recall accuracy is associated with the presence of more perceptual details in the memories (Brewin, Huntley, & Whalley, 2012; Johnson, Suengas, Foley, & Raye, 1988; Schooler, Gerhard, & Loftus, 1986).

In the present study, we aimed to investigate the ability of visual and verbal cues to lead to the retrieval of images of the previously presented stimuli as part of the recall process, as well as to determine the extent to which memory accuracy was predicted separately by stimulus valence, cue type, and the occurrence of such imagery. To clarify whether such imagery was similar to the postexperimental images that have previously been reported, after completing the task, participants were required to complete involuntary memory diaries over the subsequent seven days. It was predicted that negative as compared to positive pictures and visual as compared to verbal cues would elicit imagery that both corresponded more frequently to the picture presented and was more vivid. Second, we predicted that negative as compared to positive pictures and visual as compared to verbal cues would be associated with more accurate recall, and that these effects would be moderated by the presence of imagery. Finally, in accordance with research that has employed films differing in valence (Arnaudova & Hagenaars, 2017), as well as with theories of posttraumatic stress disorder, we predicted that both negative images and images that had been prompted by a visual cue would come spontaneously to mind more often over the following week.

## Method

### Participants

The participants were 40 undergraduate students (30 female, 10 male) from 18 to 23 years of age (mean = 19.60, *SD* = 1.41). They were volunteers or received course credits in order to take part.

### Materials and measures

#### Visual scenes

Thirty scenes^1^ from the International Affective Pictures System (IAPS) (Lang, Öhman, & Vaitl, 1988) were chosen on the basis of their affective valence rating, which ranged from 1 (*very unpleasant*) to 9 (*very pleasant*). The positive scenes (mean = 7.20) and negative scenes (mean = 2.30) did not differ in arousal (means = 5.11 and 5.69, respectively), *t*(28) = 2.03, *p* > .05. Two additional IAPS pictures were used to illustrate the procedure to participants before the study began.

#### Retrieval cues

**Fig. 1:**
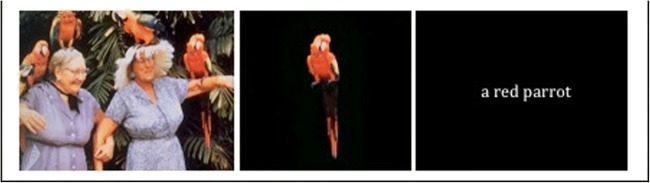
A positive scene, with its corresponding visual and verbal cues

For each scene, two matched cues were created, one visual and one verbal, representing the same neutral object or background feature (see Fig. 1 for an example; a complete list of the scenes and cues is given in Supplementary Table 1). Participants saw one cue corresponding to each scene, depending on their group assignment (see the Design and Procedure section). Each cue was unique to its corresponding scene. Photoshop CS5 was used to create the visual cues, which involved removing parts of the background or foreground or cutting out parts of the scene to be presented in isolation on a black background. The verbal cues were presented in Times New Roman 72-point font in white on a black background. All cues were presented at the center of the screen.

A separate sample of ten participants rated the cues for valence on the IAPS 1–9 scale, in the absence of their surrounding context; they rated both the verbal (mean = 5.10) and visual (mean = 5.15) cues as being neutral. To check that the visual cues were rated as equally neutral within positive and negative picture contexts, a further sample of ten participants rated the cues in different contexts and on the same valence scale, reporting averages of 4.77 for cues appearing in positive pictures and 4.97 for cues appearing in negative pictures. Finally, to check that the visual cues were not more strongly related to the content of the positive than of the negative pictures (or vice versa), this same sample rated the cues for thematic relatedness on a similar scale, ranging from 1 (*not at all thematically related*) to 9 (*extremely thematically related*). These ratings averaged to 3.41 for cues appearing in positive pictures and 2.97 for cues appearing in negative pictures.

#### Distraction task

Classical music was played for 30 s, and participants were required to report when a new instrument was introduced and the name of that instrument. This task was designed to prevent rehearsal of the final scenes. The music was a section from *The Planets* by Gustav Holst, “Jupiter, the Bringer of Jollity.”

#### Rating of vividness of imagery

Participants rated the vividness of any visual imagery using a scale adapted from the Vividness of Visual Imagery Questionnaire: 1 = *no image came to mind*, 2 = *a dim and vague image came to mind*, 3 = *a reasonably clear image came to mind*, 4 = *an image as clear as day came to mind* (Marks, 1973).

#### Rating of accuracy

Two researchers rated the accuracy of participants’ picture recall using the following scale: 1 = *no details were correct*, 2 = *a few details such as the scene valence or colors were correct*, 3 = *most details were correct but errors were present*, 4 = *all the details were correct*. One researcher rated accuracy during the experiment, and the second researcher rated it after the participants’ responses had been transcribed. The ratings were highly consistent (weighted kappa = .95).

#### Memory diary

For the following seven days, participants kept a paper-and pencil diary in which they recorded any spontaneously occurring memories or images of the stimuli seen in the experiment. They were instructed to complete the diary at the same time each day, recording any intrusion experienced that day. To distinguish such images from deliberate attempts to recall the stimuli, participants were giving the definition of an involuntary memory or image as one that “comes to mind spontaneously without any conscious attempt to bring it to mind.” As well as recording what the image consisted of, they used the vividness scale employed during the experiment to rate each intrusion. The methodology was similar to that used in the vast majority of previous trauma-film studies (James et al., 2016).

### Design and procedure

The mixed-model design involved two within-subjects variables: the valence of the scene (positive or negative) and the type of cue (visual or verbal). All participants viewed the same mixture of positive and negative scenes, with half the scenes of each valence (i.e., seven or eight) having a visual, and the remainder a verbal, retrieval cue. We used one between-subjects variable (participants were randomly assigned to one of two conditions so that the assignment of cue types to scenes was counterbalanced). The dependent variables were accuracy in the cued recall task as well as the number and vividness of images that occurred, both at recall and during the following week.

For this intentional memory task, participants were informed that they would be required to view and later to recall pictures that could depict some very negative and distressing scenes. Two demonstration pictures, one positive and one negative, were shown to participants in order to allow them to decide whether they wanted to take part in the study. None withdrew at this stage. Each of the 30 scenes was presented in randomized order on a computer screen for 10 s, both preceded and followed with a black screen for 0.5 s. Following the distraction task, participants were told that they would be shown reminders of the previously seen pictures and that these would be either descriptions or parts of the previous pictures. The reminders were chosen to be neutral background objects or features, for two reasons: first to ensure that participants had fully encoded the scenes as instructed, and not simply focused on the most salient aspects, and second, to ensure that the comparison of visual versus verbal cues could not be influenced by the valence of the retrieval cue. Participants viewed the randomly ordered visual and verbal cues for 10 s, described as many details of the corresponding picture as they could, and then reported whether an image had been prompted by the cue and how vivid it was.

The diary was then described, and participants were told to start completing it on the same day on which the experiment began. Participants provided cell phone numbers so they could be reminded each day at 7 p.m. by text message to complete the diary. The diary was returned after seven days.

## Results

### Number and vividness of retrieved images

All participants reported at least some images coming to mind following the cue presentations. The average number per person was 16.18 images that correctly corresponded to the original picture (i.e., for just over half the cues) and 2.68 incorrect images.^2^ Table 1 presents the average vividness and number of images elicited by the visual and verbal cues corresponding to positive and negative pictures. A 2 (Valence: positive vs. negative) × 2 (Cue Type: visual vs. verbal) analysis of variance (ANOVA) on vividness ratings indicated a significant effect of valence, *F*(1, 35) = 7.95, *p* = *.*008, *η*_p_^2^ = .18, whereby negative images were more vivid than positive images. There was no main effect of cue type, *F*(1, 35) = .11, *p* = .74, and no interaction between valence and cue type, *F*(1, 35) = 2.72, *p* = .11.

**Table 1:** Mean number and vividness of images (with standard deviations) retrieved during cued recall

Valence	Cue	Mean vividness	Number of correct images	Number of incorrect images
Positive	Visual	3.18 (0.47)	4.22 (1.51)	0.85 (0.92)
	Verbal	3.04 (0.60)	3.30 (1.74)	0.85 (0.89)
Negative	Visual	3.31 (0.43)	4.82 (1.58)	0.20 (0.40)
	Verbal	3.40 (0.65)	3.22 (1.67)	0.77 (0.77)

A similar ANOVA on the numbers of images that correctly corresponded to the original pictures (i.e., that received an accuracy rating greater than 1) revealed a main effect of cue, *F*(1, 39) = 47.82, *p* < .001, *η*_p_^2^ = .55, indicating that visual cues elicited more images than did verbal cues. We found no main effect of valence, *F*(1, 39) = 2.39, *p* = .13, and no cue type by valence interaction, *F*(1, 39) = 1.54, *p* = .22. The corresponding ANOVA on the numbers of incorrect images indicated main effects of cue, *F*(1, 39) = 4.61, *p* < .038, *η*_p_^2^ = .11, and of valence, *F*(1, 39) = 12.06, *p* < .001, *η*_p_^2^ = .24, as well as a two-way interaction, *F*(1, 39) = 6.46, *p* = .015, *η*_p_^2^ = .14. As is shown in Table 1, incorrect images were more infrequent after negative pictures elicited by visual cues than after any other valence–cue type combination.

### Accuracy of cued recall

No significant differences were found for the average accuracy of cued recall in the two counterbalanced conditions, *t*(59) = 1.72, *p* = .78, and these were collapsed for the analyses. The accuracy data are shown in Table 2. A 2 (Valence: positive vs. negative) × 2 (Cue Type: visual vs. verbal) × 2 (image vs. no image) ANOVA revealed main effects of valence, *F*(1, 31) = 7.98, *p* = .008, *η*_p_^2^ = .20; cue type, *F*(1, 31) = 8.84, *p* = .006, *η*_p_^2^ = .22; and presence of an image, *F*(1, 31) = 901.54, *p* < .001, *η*_p_^2^ = .97, indicating that accuracy was greater for negative scenes, visual cues, and when images were retrieved.

**Table 2:** Mean accuracy (with standard deviation) of recall, by valence, cue type, and image

Valence	Cue	Mean accuracy with image	Mean accuracy without image
Positive	Visual	2.90 (0.49)	1.02 (0.08)
	Verbal	2.74 (0.77)	1.03 (0.13)
Negative	Visual	3.36 (0.41)	1.08 (0.22)
	Verbal	2.90 (0.68)	1.03 (0.95)

The analysis also revealed several significant interactions, including one between valence and cue, *F*(1, 31) = 4.89, *p* = .035, *η*_p_^2^ = .14. Pairwise comparisons indicated that in the presence of visual cues, negative scenes were recalled more accurately than were positive scenes, *t*(31) = 3.91, *p* < .001. In the presence of verbal cues, there were no differences in recall accuracy, *t*(31) = 0.20, *p* > .80. Another interaction was found between valence and retrieval of an image, *F*(1, 31) = 6.35, *p* = .017, *η*_p_^2^ = .17. Pairwise comparisons indicated that in the presence of an image, the advantage for recall of negative over positive scenes was significant, *t*(31) = 2.09, *p* < .05, but in the absence of an image there was no effect of valence, *t*(31) = 0.95, *p* > .30.

The final significant interaction was that between cue type and retrieval of an image, *F*(1, 31) = 8.02, *p* = .008, *η*_p_^2^ = .20. Pairwise comparisons indicated that in the presence of an image, the recall advantage conferred by visual cues was significant, *t*(31) = 3.06, *p* < .01, but in the absence of an image there was no effect of cue type, *t*(31) = 0.11, *p* > .90. The three-way interaction was not significant, *F*(1, 31) = 1.71, *p* = .201.

### Diary study of involuntary memories

Twenty-nine participants returned diaries, and all recorded at least one involuntary visual memory of the test material during the seven-day follow-up period, with a mean of 6.79 (*SD* = 3.96) memories reported (including repeated memories of the same scenes). The number of involuntary memories ranged from 1 to 15 (one participant reporting 23 memories was excluded as an outlier). Out of 193 memories, 164 (85%) had previously come to mind during the recall phase of the experimental session. Table 3 presents the average numbers of involuntary memories for positive and negative scenes that had been prompted at initial recall by visual and verbal cues.

**Table 3:** Means (with standard deviations) of the number of involuntary memories reported in the daily diary

Valence	Cue	Number of involuntary memories
Positive	Visual	0.82 (1.22)
	Verbal	0.82 (0.98)
Negative	Visual	3.75 (3.07)
	Verbal	1.39 (1.52)

A 2 (Valence: positive vs. negative) × 2 (Cue Type: visual vs. verbal) ANOVA revealed main effects of valence, *F*(1, 27) = 24.83, *p* < .001, *η*_p_^2^ = .48, and cue type, *F*(1, 27) = 10.39, *p* = .003, *η*_p_^2^ = .28, qualified by a two-way interaction, *F*(1, 27) = 12.49, *p* < .001, *η*_p_^2^ = .32. Analysis of the interaction revealed that of the pictures initially retrieved after visual cues, negative scenes were more likely than positive scenes to be involuntarily recalled, *t*(27) = 4.83, *p* < .001. Among the negative pictures, those initially retrieved after visual cues were more likely to be involuntarily recalled than were those retrieved after verbal cues, *t*(27) = 3.61, *p* = .001. There were no significant differences between the numbers of positive scenes that were involuntarily recalled after initial exposure to visual and verbal cues, *t*(27) = 0.00, *p* = 1.00, or between the numbers of positive and negative scenes that were involuntarily recalled after initial exposure to verbal cues, *t*(27) = 1.79, *p* = .084.

## Discussion

Our first prediction was that negative as compared to positive pictures and visual as compared with verbal cues would elicit imagery that both corresponded more frequently to the picture presented and was more vivid. This prediction was only partially supported: Relative to positive scenes, negative scenes did not elicit a greater number of correct images, but their images were more vivid. In contrast, visual cues elicited more correct images, but there was no increase in vividness. The combination of a negative scene and a visual cue resulted in fewer incorrect images being elicited.

The phenomenological experience of imagery accompanying retrieval was present in all participants and occurred on average to over half of the available cues. This task-related imagery is distinct from the associated autobiographical memories that have been prompted by previous experimental procedures, such as generating word associations and passively viewing words and phrases (Brewin & Soni, 2011; Mace, 2005; Schlagman & Kvavilashvili, 2008).

Our second prediction was that negative as compared to positive pictures and visual as compared with verbal cues would be associated with more accurate recall, and that these effects would be moderated by the presence of imagery. Our data replicated previous studies that had found negative stimuli to be recalled more accurately than positive stimuli (Kensinger, 2009; Ochsner, 2000). Furthermore, we showed that the recall advantage for negative scenes was limited to instances in which an image was retrieved or a visual cue had been used (i.e., a cue associated with an increased probability of imagery retrieval). In the absence of these elements, we observed no advantage in accuracy, just as there was no advantage in accuracy for a visual cue unless it was followed by the retrieval of an image.

Although it is theoretically possible for the scenes also to have been represented in a verbal form, in practice the absence of an image resulted in a very limited account of its content being given, with recall scores being very close to their floor. The actual size of the advantage conferred by an image was hard to estimate in this study, because of the use of accuracy ratings rather than a count of numbers of specific details. It seemed from the mean ratings, however, that retrieved images improved recall, but by no means captured all the details available in the original scene.

The large effect size for recall accuracy associated with retrieval of an image echoes other findings concerning long-term memory. For example, memory for the colors of 232 objects seen one after the other for 1 s apiece was compared with memory for the colors of three objects held in working memory for 3 s (Brady et al., 2013). Although the color of around half of the 232 objects was forgotten, the fidelity with which color was recalled for remembered items was equivalent to that for the three items in working memory. In another study, patients with posttraumatic stress disorder were better able to discriminate words and phrases from their own trauma narrative, as compared to another trauma narrative, when those words or phrases elicited an involuntary visual image (Brewin et al., 2012). As in that study, we usually found the images that came to mind to be appropriate to the retrieval cue, but on some occasions to be incorrect.

One caveat involves the difficulty of matching the strengths of verbal and visual cues in an experiment, as well as of determining the strength of a visual cue for a real-world event. Rather than conclude that visual cues are generally more effective than verbal cues in picture recall, therefore, our data should be seen as throwing light on one effective mechanism (image retrieval) that is likely to increase the chance of successful recall.

We agree with Brewer and Pani (1983) that these aspects of memory are potentially of great importance, even though it is very difficult to establish unequivocally that they have a causal impact. For example, it is difficult to determine the extent to which aspects of the encoding contributed both to enhanced recall and to the probability of an image being retrieved. However, the fact that these images continued to intrude over the following week, in some cases, consistent with findings from numerous previous studies (Brewin, 2014; Ferree & Cahill, 2009; James et al., 2016), affirms that participants who reported an image were unlikely to have been simply describing the recall process in another way. Rather, they were describing a phenomenological experience that has been observed outside, as well as within, the confines of the experimental session. In future studies it would be desirable to collect more detailed information on the content of these images—for example, to verify what material from the wider scene the images included beyond the cue prompts.

Our third prediction was that these later involuntary memories would be more likely to occur when the original scene was negative and had been elicited by a visual cue, and this was confirmed by the data. This more persistent imagery is likely to offer a recall advantage to negative stimuli over the longer term, although this was not explicitly tested in our study. In previous research, negative films have routinely elicited more spontaneous memories than have positive films (Arnaudova & Hagenaars, 2017). The data are also consistent with claims made by psychological theories of posttraumatic stress disorder that intrusive trauma memories are elicited more readily by sensory reminders of the traumatic event (Brewin et al., 2010; Ehlers & Clark, 2000), as well as with evidence that the degree to which stimuli evoke perceptual priming predicts the development of involuntary images (Michael & Ehlers, 2007; Sündermann, Hauschildt, & Ehlers, 2013).

Turning to the nonpredicted findings, it was intriguing that whereas subjective vividness was associated with negative versus positive stimulus valence and not with cue type, the probability of an image being retrieved was associated with cue type and not with valence. The data suggest that these two aspects of memory have different underlying mechanisms. In a study from our laboratory using a traumatic film as a stimulus, we similarly found that the vividness and frequency of involuntary memories of the film were uncorrelated. Moreover, frequency and amount of perceptual detail, but not vividness, were associated with reductions in heart rate during specific film scenes (Chou, La Marca, Steptoe, & Brewin, 2014).

The results are also reminiscent of other reports that both the subjective vividness of the recall experience and the accuracy of recall have a degree of functional independence, whether the stimuli are pictures (Sharot, Delgado, & Phelps, 2004) or words (Dougal & Rotello, 2007). There is evidence that the subjective vividness of visual imagery is dependent on the availability of working memory resources, specifically the visuospatial sketchpad (Baddeley & Andrade, 2000). These resources may not be implicated in the relative probability of an image being retrieved to a visual or verbal cue.

Our study is limited by the absence of neutral pictures and by the weak (although nonsignificant) positive association between valence and arousal. Furthermore, it should be noted that there may have been overall differences in the numbers of details that could be remembered for positive and negative scenes, potentially having an effect on the rating of participants’ recall. Nevertheless, the findings suggest that the established recall advantage for negative over positive scenes has to do with the fact that they are accompanied in the short term by more vivid imagery, and in the longer term by more persistent imagery. Our data support previous arguments that memory theories addressing effects such as that of valence on recall need to take into account the specific types of stimuli used (Brady, Konkle, & Alvarez, 2011), and pay greater attention to phenomenology (Brewer & Pani, 1983). Thus, models of recognition and recall may benefit from taking into account whether the stimuli are words, pictures, autobiographical memories, and so forth, and addressing the role of imagery retrieval.

The results may be particularly important for functional neuroimaging studies that address the processes engaged at retrieval and their relation to recall success. For example, such studies typically show that the brain regions involved in encoding sensory–perceptual detail tend to be reactivated at retrieval (Danker & Anderson, 2010) and tend to be more active during accurate than during inaccurate retrieval (Schacter & Loftus, 2013). Our finding that successful recall is often linked to the subjective experience of imagery raises the possibility that these patterns of neural activation are accounted for by retrieval of a conscious image, rather than being a more general index of memory accuracy. Assessing the content of retrieved images and discounting those that are examples of incorrect retrieval could lead to more precise ways of characterizing the neural processes underlying accuracy.

## Electronic supplementary material

Supplementary Table 1 (DOCX 1053 kb)

## Footnotes

1 Participants were originally shown 40 images, but ten were removed to make the images comparable on levels of arousal.

2 Incorrect images might theoretically have involved either scenes that were among either the 30 that were analyzed or the ten that were not analyzed. The average number of incorrect responses was very small, however, and distinguishing between error types is unlikely to affect our conclusions.
