# Supplementary material for: Imagery retrieval may explain why recall of negative scenes contains more accurate detail
Source: Mem Cognit. 2018 Oct 31;47(3):420–7. doi: 10.3758/s13421-018-0876-7 (PMC6450854; doi:10.3758/s13421-018-0876-7)
Supplement: Supplementary file 1 — (DOCX 1053 kb) [file 13421_2018_876_MOESM1_ESM.docx]

Supplementary Table 1

Positive and negative images and associated verbal and sensory cues

| Positive images | Verbal cue | Sensory cue |
| --- | --- | --- |
| Winter landscape | a bright sun | 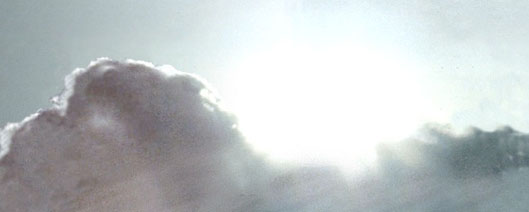 |
| Girl cooling off | a water bottle | 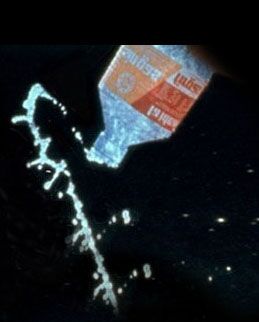 |
| Plate of food | two slices of orange | 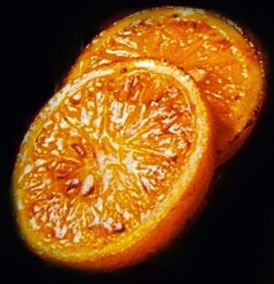 |
| Pancakes | the head of a silver fork | 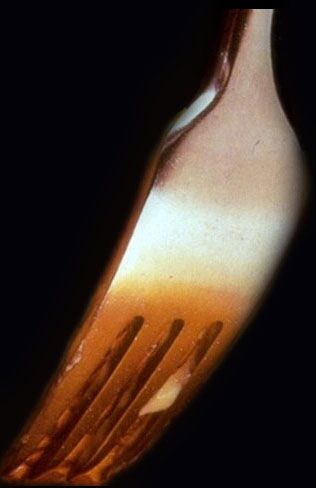 |
| Animal in tree | a tree's bark | 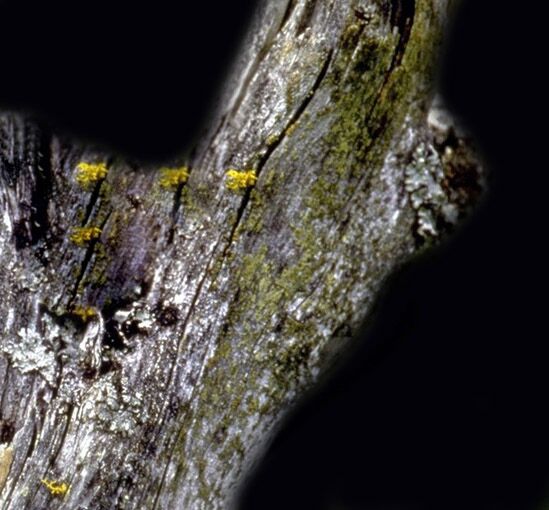 |
| Women enjoying themselves | a red parrot | 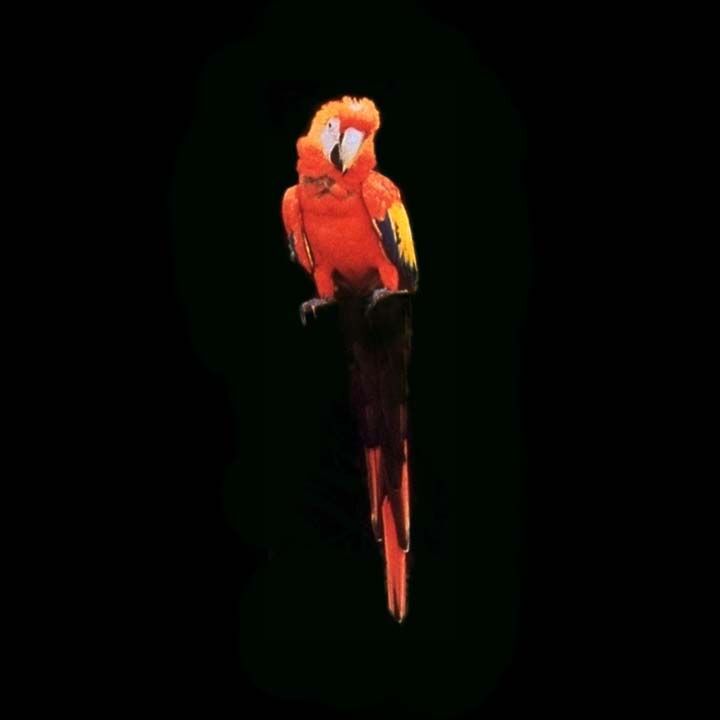 |
| Holiday boat scene | a yellow life jacket | 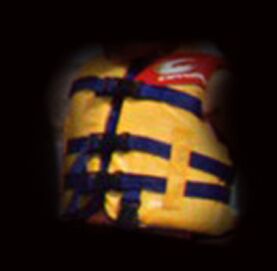 |
| Bathers | a part of a waterfall | 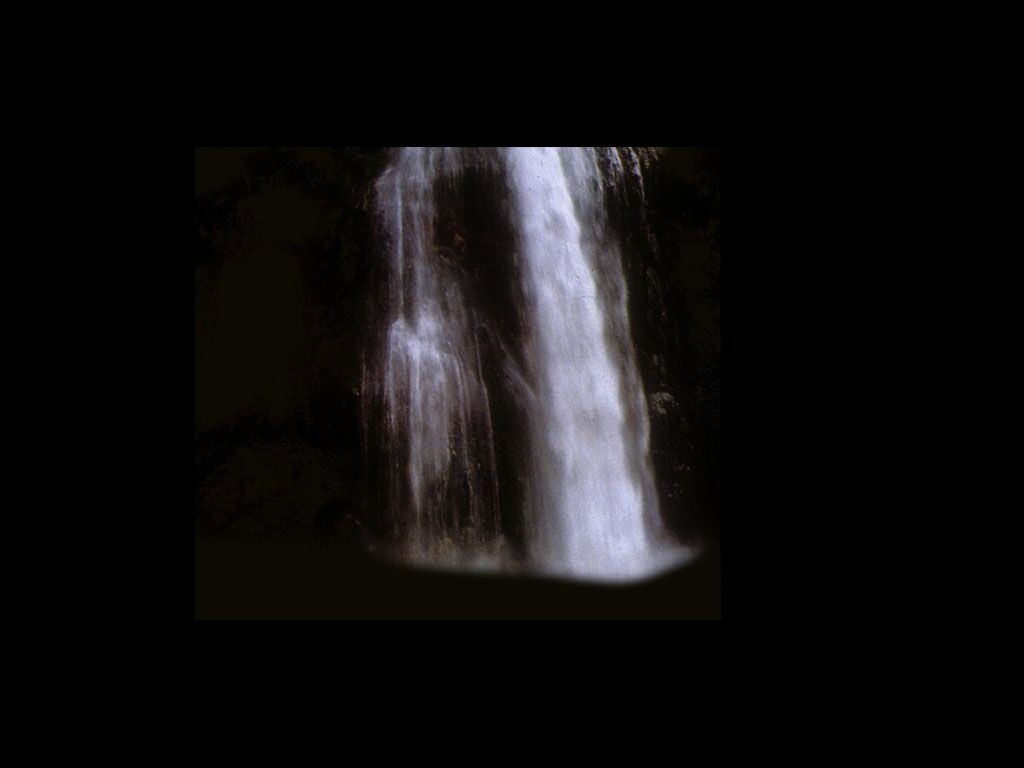 |
| Children at funfair | a yellow down sign | 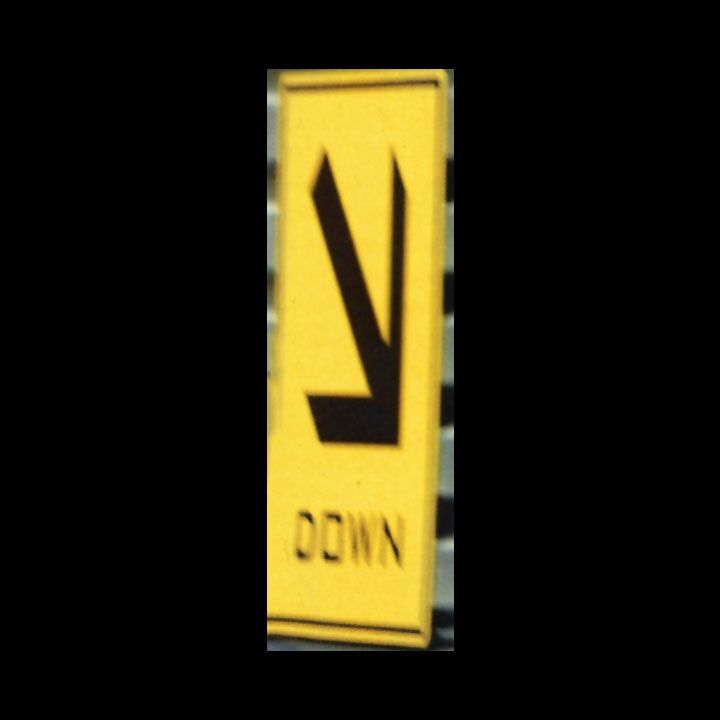 |
| Parent and children having fun | a shelf full of books | 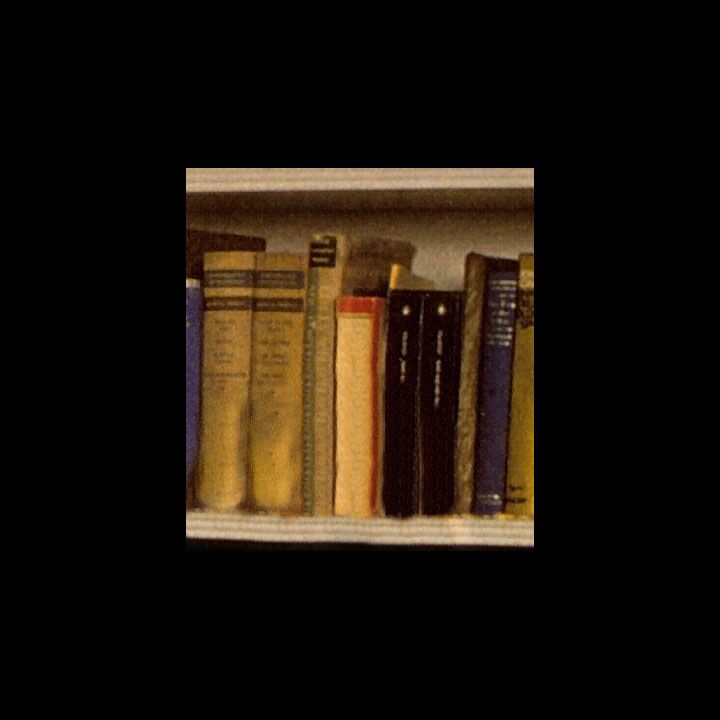 |
| Celebrating crowd | a sky full of balloons | 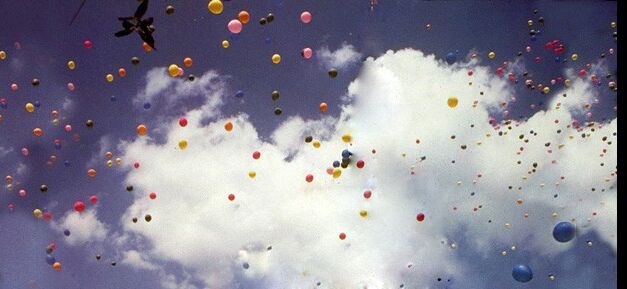 |
| Baby in bath | a running tap | 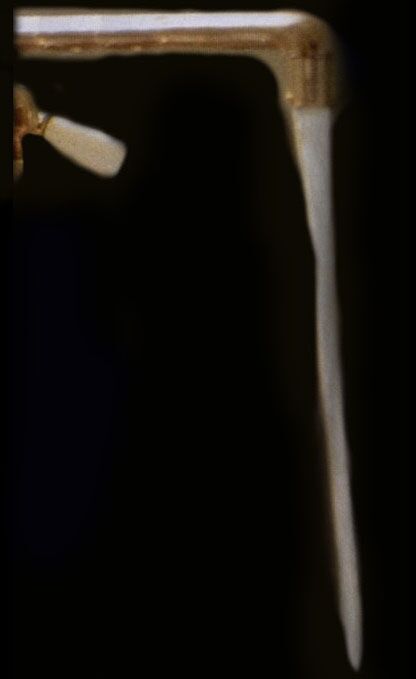 |
| Seascape with children | a sky full of seagulls | 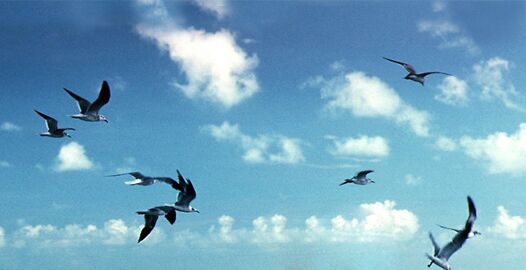 |
| Characters in theme park | a red dress with white dots | 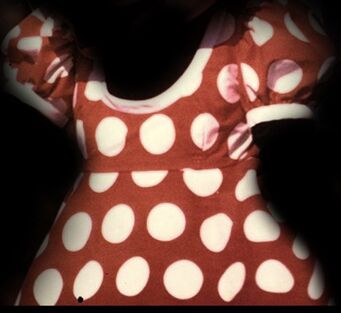 |
| Wedding scene | a bunch of flowers | 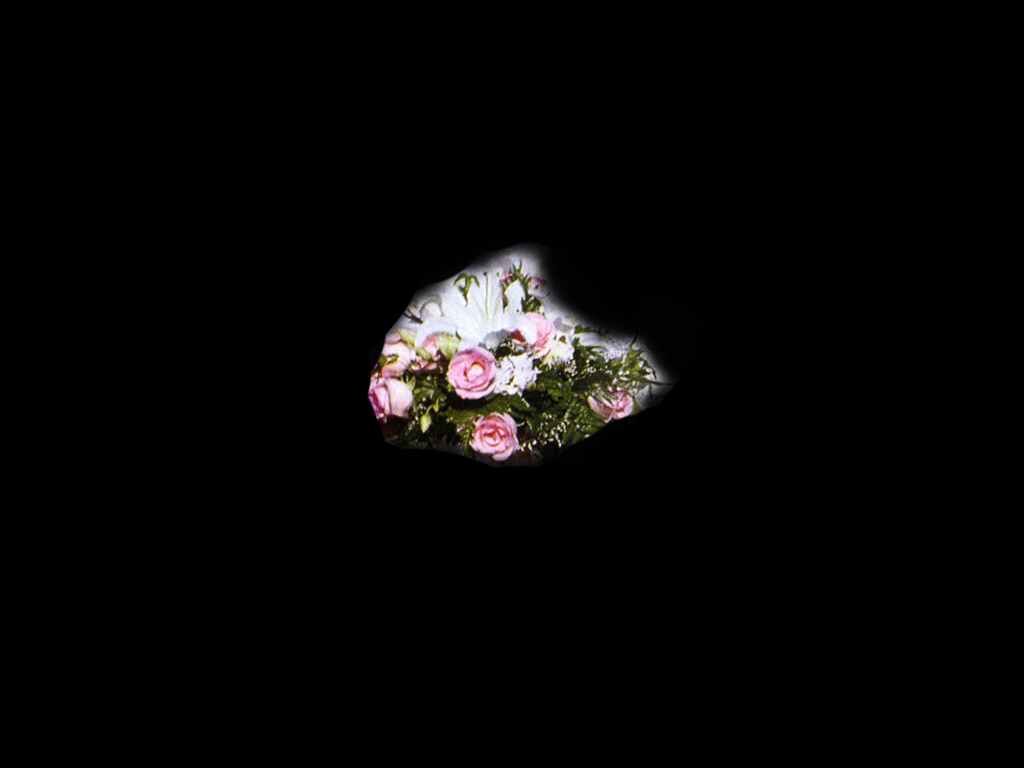 |

| Negative images | Verbal cue | Sensory cue |
| --- | --- | --- |
| Graves in forest | a green forest floor | 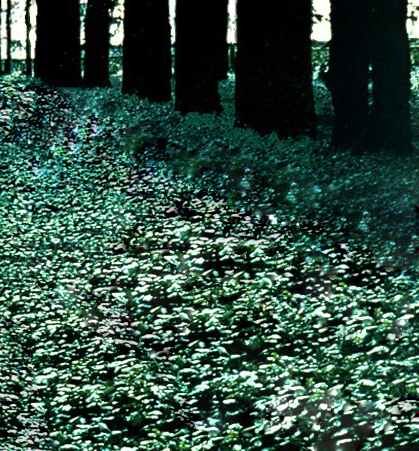 |
| Burning house | the back view of a fireman | 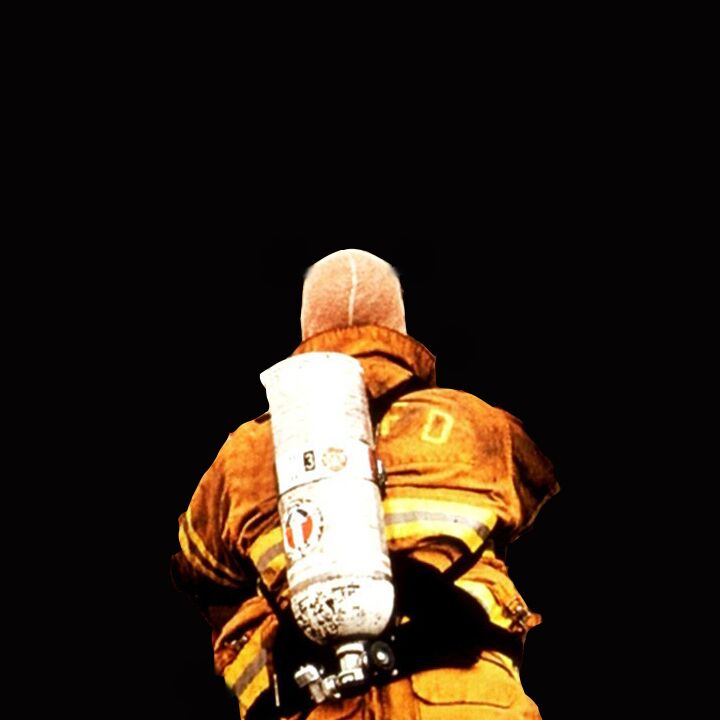 |
| Injured man | a mud path through grass | 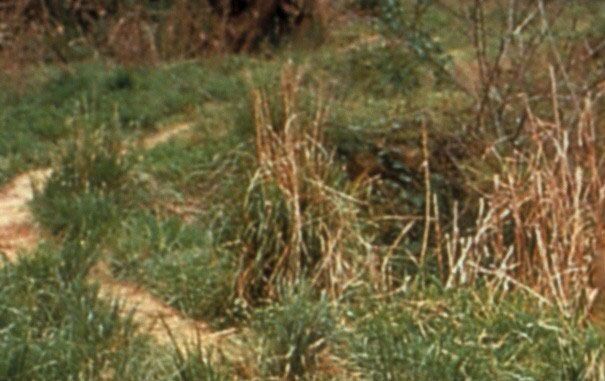 |
| Boy with deer’s head | a white wellington boot | 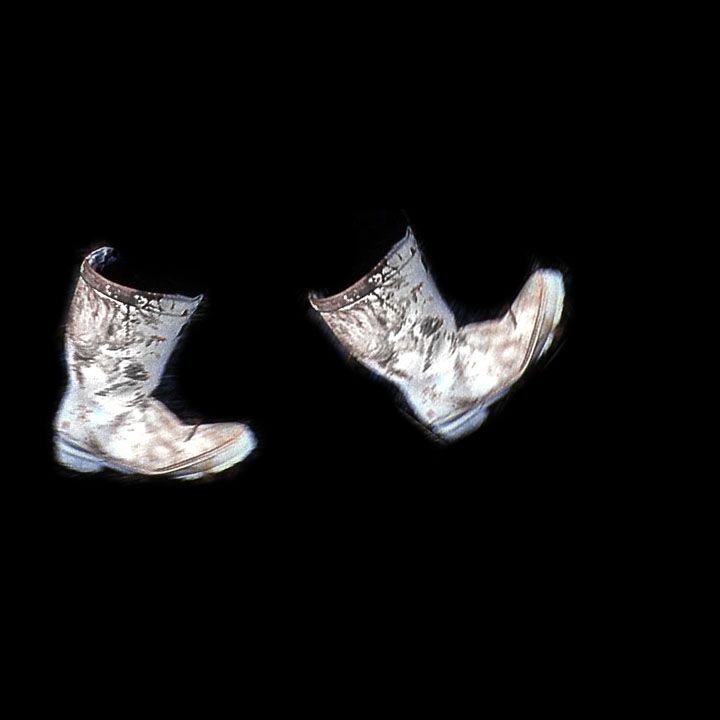 |
| Injecting arm | two white latex gloves | 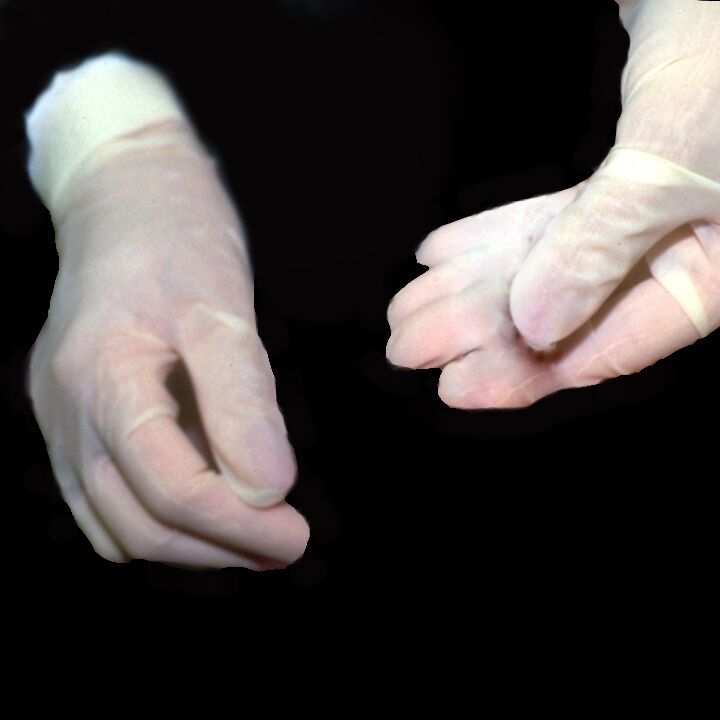 |
| Suicide scene | the back of a man's head | 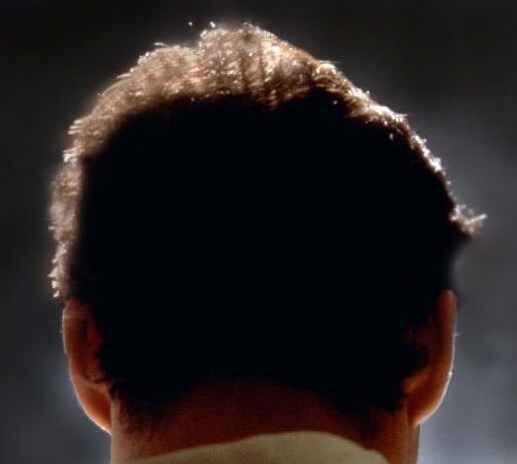 |
| Corpse | a hole full of stones and rubble | 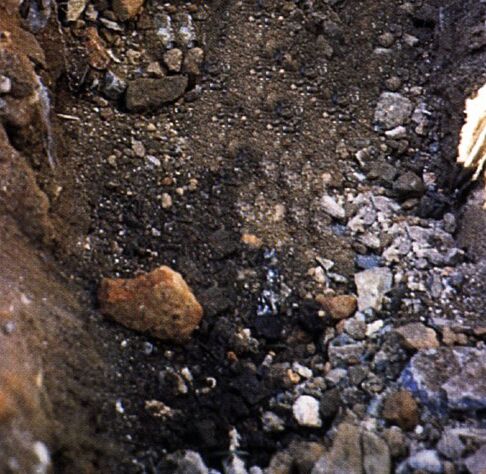 |
| Woman with facial injuries | a red jumper | 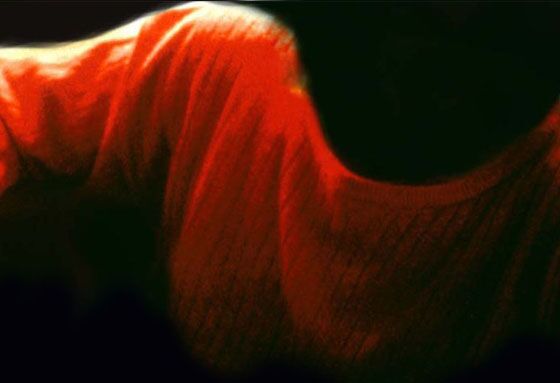 |
| Crowd with dead body | a crowd of men | 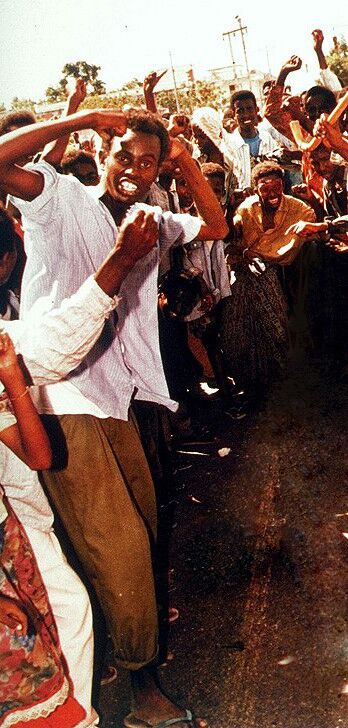 |
| Body on ground | a line of feet | 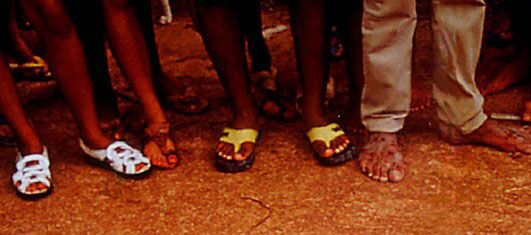 |
| Dirty lavatory | a newspaper | 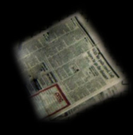 |
| Gang violence | a stationary car | 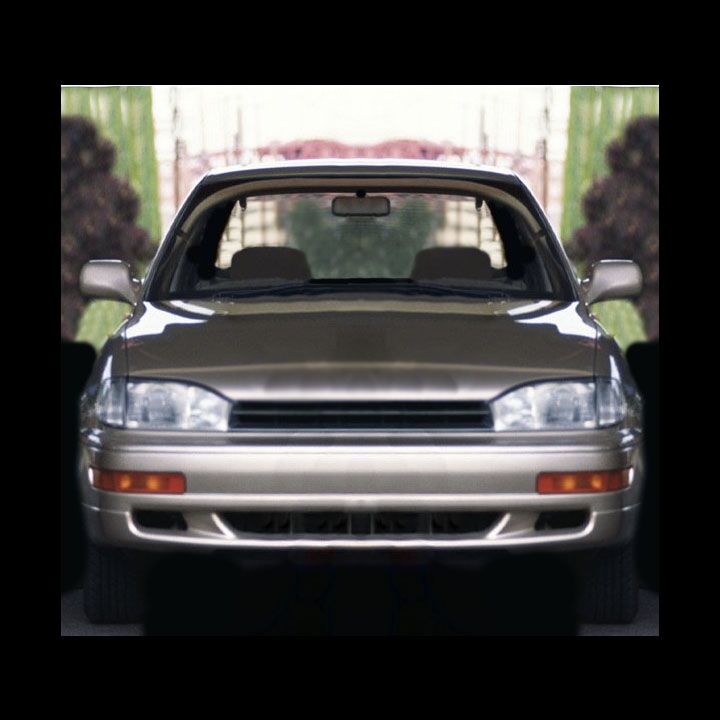 |
| Injury with blood | a blue stripey pillow | 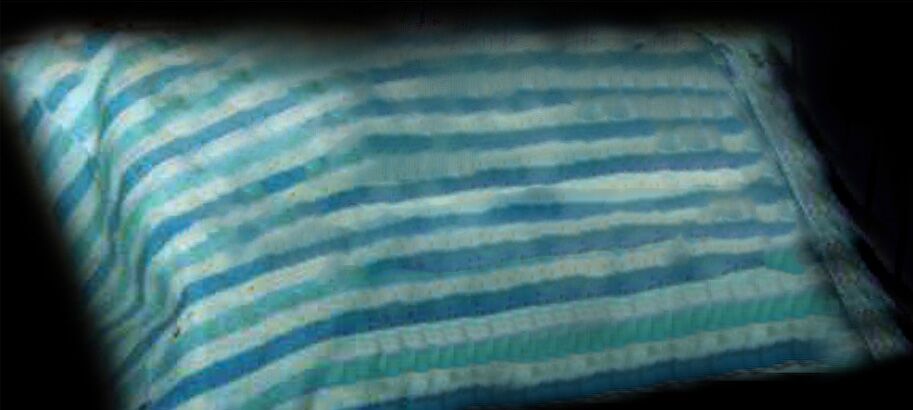 |
| Dead animal | yellow and white road markings | 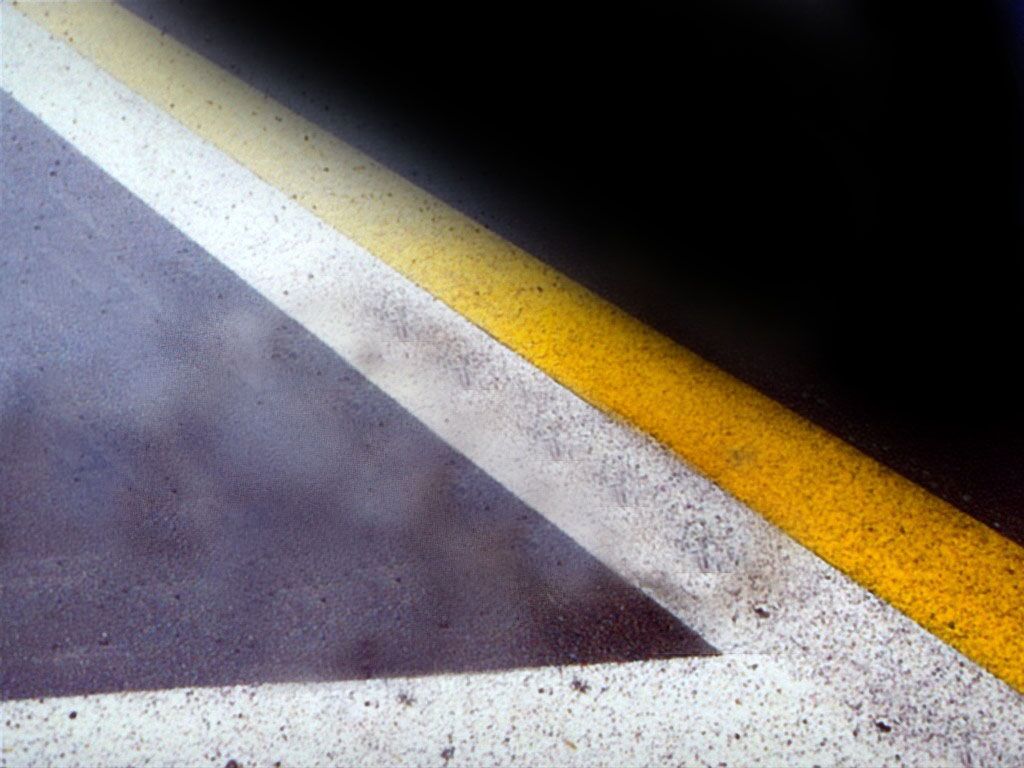 |
| Injured hand | a bowl full of red liquid | 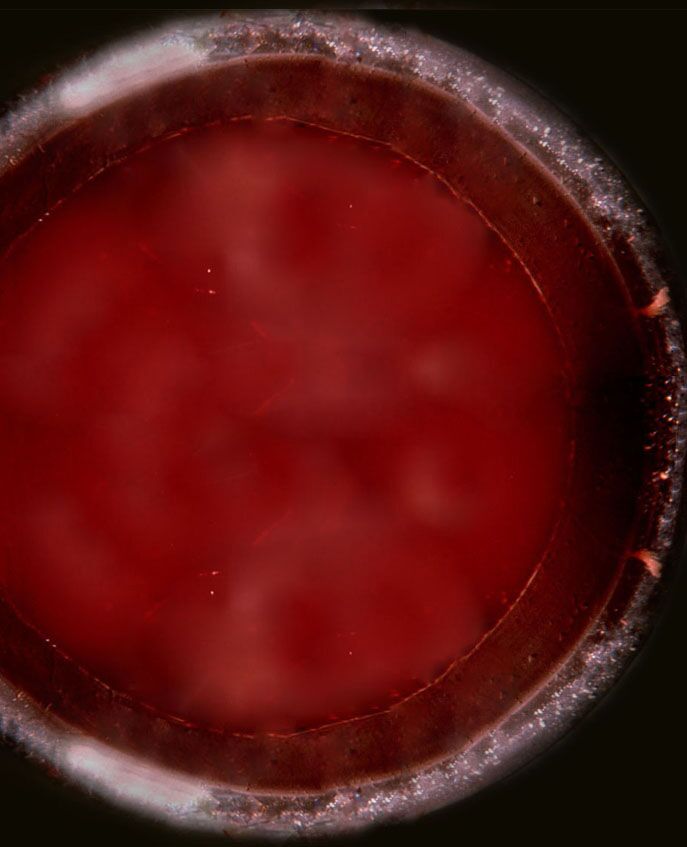 |
